# Shenling Baizhu Powder Alleviates TNBS-Induced Colitis in Rats by Improving Intestinal Epithelial Permeability and Inhibiting Inflammation Through the TLR5/MyD88/NF-κB Pathway

**DOI:** 10.3389/fphar.2022.883918

**Published:** 2022-04-28

**Authors:** Kehan Rao, Shumin Qin, Yuanming Yang, Kai Zhan, Haomeng Wu, Huan Zheng, Shaogang Huang

**Affiliations:** ^1^ Second Clinical Medical College, Guangzhou University of Chinese Medicine, Guangzhou, China; ^2^ The Second Affiliated Hospital of Guangzhou University of Chinese Medicine, Guangzhou, China; ^3^ Guangdong Provincial Key Laboratory of Clinical Research on Traditional Chinese Medicine Syndrome, Guangzhou, China; ^4^ State Key Laboratory of Dampness Syndrome of Chinese Medicine, The Second Affiliated Hospital of Guangzhou University of Chinese Medicine, Guangzhou, China; ^5^ Guangdong-Hong Kong-Macau Joint Lab on Chinese Medicine and Immune Disease Research, Guangzhou, China; ^6^ Collaborative Innovation Team of Traditional Chinese Medicine in Prevention and Treatment of Functional Gastrointestinal Diseases, Guangzhou, China; ^7^ Dongguan Hospital of Guangzhou University of Chinese Medicine, Dongguan, China

**Keywords:** shenling baizhu powder, intestinal barrier, MUC2, ulcerative colitis, TLR5

## Abstract

**Aim of the study:** To evaluate the protective effect and mechanism of shenling baizhu powder (SBP) on TNBS-induced colitis.

**Methods:** Rats were given TNBS to establish the model of colitis and subsequently treated with different doses of SBP or mesalamine (MES). In addition, the expression of the TLR5/MyD88/NF-κB signaling pathway and critical targets of the intestinal mucosal barrier was detected by immunochemical analysis techniques.

**Results:** SBP significantly ameliorated the symptoms of TNBS-induced colitis in rats and reduced the secretion of pro-inflammatory cytokines. SBP could effectively strengthen epithelial barrier integrity in TNBS-induced colitis by increasing the secretion of mucin and tight junction and inhibiting apoptosis. Furthermore, we identified the crucial role of the TLR5/MyD88/NF-κB signaling pathway in exerting the therapeutic effect of SBP.

**Conclusion:** The results of our study suggest that SBP has therapeutic effects on TNBS-induced colitis and potential value in treating and maintaining remission of colitis.

## Introduction

As the major type of inflammatory bowel disease (IBD), ulcerative colitis (UC) is characterized by mucosal inflammation initiating in the rectum and extending proximally in the colon with a high incidence rate (Ananthakrishnan et al., 2020). The risk of conversion therapy and perioperative complications is exceptionally high, and the long-term maintenance of poor relief increases the risk of colorectal cancer (Peyrin-Biroulet et al., 2016; Ungaro et al., 2019). Although various studies suggested that a dysregulated immune response, intestinal epithelial injury, gut microbiota, genetic susceptibility, and environmental factors contribute to the morbidity and development of UC, the exact pathogenesis of UC is not entirely understood.

Amino salicylates, antibiotics, biological agents (anti-TNF monoclonal antibodies), and corticosteroids are widely used in clinical settings. However, the adverse effects, financial burden, and high recurrence rate of these drugs directly limit their usage (Kobayashi et al., 2020). Therefore, complementary and alternative therapy has been an attention-grabbing issue for UC patients, clinicians, and researchers.

Many Chinese herbal formulas or plant extracts have attracted increased attention for use in the treatment and maintain remission owing to their effects and low toxicity (Yang et al., 2021). Among them, shenling baizhu powder (SBP) is a classic prescription of traditional Chinese medicine for diarrhea and abdominal pain, mainly including related components (Table 1). SBP chemical component analysis has been reported in many works (Ji et al., 2019; Wang et al., 2021). Herbs that make up SBP, or the main components of these herbs, have been proved to regulate gut microbiota, inhibit inflammation, and repair the intestinal mucosal barrier (Table 2). A recent study showed that SBP significantly decreased the serum Th1 in elderly patients with ulcerative colitis complicated by bloody purulent stool (Li et al., 2021). In addition, several clinical studies have demonstrated the safety of SBP in the treatment of patients with irritable bowel syndrome, chronic obstructive pulmonary disease, type 2 diabetes, and lung cancer (Huang et al., 2019; Jiang et al., 2021; Mao et al., 2021; Shi et al., 2021).

**TABLE 1:** Recipe of SBP decoction.

Components	Vernacular name	Ratio	Batch code
*Panax ginseng* C.A.Mey [Araliaceae]	Renshe	13	19101781
*Poria cocos* (Schw.)Wolf [Polyporaceae]	Fulin	13	19100891
*Atractylodes macrocephala* Koidz [Asteraceae]	Baizhu	13	19082541
*Paeonia lactiflora* Pall [Paeoniaceae]	Shanyao	13	19101751
*Lablab purpureus* subsp. purpureus [Fabaceae]	Baibiandou	11	19071201
*Nelumbo nucifera* Gaertn [Nelumbonaceae]	Lianzi	6	19060961
*Coix lacryma-jobi* L. [Poaceae]	Yiyiren	6	19090431
*Wurfbainia villosa* (Lour.) Skornick & A.D.Poulsen [Zingiberaceae]	Sharen	6	19080351
*Platycodon grandiflorus* (Jacq.) A.DC [Campanulaceae]	Jiegeng	6	19091931
*Glycyrrhiza glabra* L. [Fabaceae]	Gancao	13	19111271
*Citrus × aurantium* L. [Rutaceae]	Chenpi	6	19110851

**TABLE 2:** Pharmacological activities of SBP.

Components	Effect
Platycodin D	Platycodin D increases the level of anti-inflammatory cytokines and alters macrophage proportions Guo et al. (2021)
Atractylenolide	Atractylenolide I stimulate intestinal epithelial cell migration and proliferation. Atractylenolide III ameliorates intestinal inflammation in mice by reducing oxidative stress and regulating intestinal microbiota Song et al. (2017), Ren et al. (2021)
Dolichos lablab L.	Dolichos lablab L. selectively stimulates the growth of beneficial bacteria and markedly decreases the mast cell count and tumor necrosis factor-α level in the colon Chun et al. (2018), Myint et al. (2018)
Adlay	Adlay have the effect of anti-tumor, anti-inflammatory activity, and anti-metastatic activity Guo et al. (2015)
Ginsenoside	Ginsenoside Rk3 could bring beneficial alterations in gut microbiota diversity and repair the intestinal barrier dysfunction. Ginsenoside Rb1 could promote intestinal epithelial wound healing against intestinal mucosal damage Toyokawa et al. (2019), Bai et al. (2021)
Amomum villosum	The therapeutic effect of Amomum villosum is associated with attenuating the expression level of pro-inflammatory cytokines, promoting Treg differentiation and function, rebalancing CD4+ T-cell subsets, and increasing the diversity of dominant bacteria Chen et al. (2018)
Carboxymethyl pachyman,CMP	CMP alleviates colon injury and regulates the ecological balance of the intestinal flora.CMP exhibits immune-regulatory, anti-inflammatory, and antioxidant activities Wang et al. (2018)

Dysregulation of innate immune response by Toll-like receptors (TLRs) is a significant feature of UC (Rakoff-Nahoum et al., 2006). TLR5 is an essential member of TLRs and is highly expressed in the colonic epithelial cells (Price et al., 2018). The mucus layers of epithelial TLR5-deficient mice were widely colonized by symbiotic microorganisms, resulting in spontaneous colitis (Burgueño and Abreu, 2020). MUC2 is a mucinous protein expressed only in the intestinal epithelium, which is secreted by goblet cells, polymerizes to generate the dense net-like structures of the inner mucus layer, and is an essential barrier to ensuring that commensal bacteria do not induce an inflammatory response by the organism (Johansson et al., 2011). Studies found that the bacteria-free mucous layer no longer covers the colonic epithelium of MUC2-deficient mice (Zarepour et al., 2013), and the expression of MUC2 and goblet cells in the colonic mucosa were significantly reduced in UC patients with a thinner and penetrable mucus layer (Johansson et al., 2014). Previous research suggested that increasing the intake could reduce the MUC2 mucus barrier consumption by the microbiota (Tian et al., 2021). *Bifidobacterium dentium* downregulated TNBS-driven colonic inflammation by increasing goblet cell remodeling and MUC2 secretion (Engevik et al., 2021). Additionally, flagellin/TLR5 response is confined to the basolateral normally and not apically. However, when the colonic epithelial integrity is impaired TLR5 could be exposed to bacterial flagellin, triggering TLR5-mediated inflammation (Letran et al., 2011). Multiple studies discovered that TLR5 gene polymorphisms were distinctly associated with UC in humans and animals (Kathrani et al., 2010; Meena et al., 2015). While the precise mechanism by which loss of TLR5 and MUC2 promotes inflammatory bowel diseases remains under investigation.

In this study, we further investigate the relationship between the destruction of the MUC2-related mucus barrier and the colonic inflammation triggered by the TLR5/MyD88/NF-κB pathway in the TNBS-induced colitis model. On this basis, the efficacy and mechanism of SBP in the treatment of colitis were further discussed.

## Materials and Methods

### Drug Preparation

The detailed composition and ratio of SBP are shown in Table 1, which was according to the recommendations of Pharmacopoeia of the People’s Republic of China (2020 edition). All the raw herbal granules were purchased from Jiangyin Tianjiang Pharmaceutical Industry Co., Ltd. (Jiangsu, China) Calculated with reference to the 60 kg adult body weight and rats equivalent dose formula, three gavage doses (2.95, 5.9, and 11.8 g/kg) were configured in distilled water. The mesalamine (MES) SR granules (Lot number H20143164, Shanghai Ethypharm Co., Ltd., Shanghai, China) were dissolved in distilled water to a concentration of 0.04 g/ml.

### TNBS-Induced Colitis and Animal Experiments

The male Wistar rats were purchased from the Experimental Animal Center of Guangzhou Southern Medical University (SCXK(Yue)2016-0041). The animal protocol was approved by the Research Institute of the Animal Protection and use Committee of Guangdong Provincial Hospital of Traditional Chinese medicine [SCXK(Yue) 2018-0094]. After 7 days of adaptive feeding, all the rats were randomly divided into 6 groups according to random number table method: control (*n* = 6), TNBS + PBS group (*n* = 6), low-dose SBP group (2.95 g/kg, *n* = 6), medium-dose SBP group (5.9 g/kg, *n* = 6), high-dose SBP group (11.8 g/kg, *n* = 6), and MES group (50 mg/kg, *n* = 6). In all the groups except for the normal control group, acute UC was induced using one administration of 2,4,6-Trinitrobenzenesulfonic acid (TNBS, P2297-10 ml, Sigma-Aldrich). The specific operation was as follows: TNBS (100 mg/kg) was prepared in 25% ethanol in equal length and then injected into the colon 8 cm through the infant catheter, and the rat tail was lifted upside down for 30 s to ensure that the solution was evenly distributed in the colon. The control group was subjected to a PBS solution instead. The rats in each group were given intragastric administration or normal saline for 14 days. Finally, the serum and colon were collected in a sterile frozen tube and stored at −80°C for follow-up testing.

### Evaluation of Clinical Scoring

The colon length and mucosal morphology were measured and photographed in each rat. DAI was evaluated using three factors: the percentage decrease in body mass, fecal viscosity, and bleeding status (Supplementary Table S1). The morphological changes in intestinal mucosa such as hyperemia, edema, and ulcer were scored by colon mucosal damage index (CMDI). The scoring criteria were listed in Supplementary Table S2.

### HE Staining and Alcian Blue-Periodic Acid-Schiff Staining of the Colon Tissues

The colon tissues were fixed with 4% paraformaldehyde for 24 h. They were embedded in paraffin, cut into 3 μM sections, stained with hematoxylin-eosin, and the colon tissue injury was observed under a high-magnification microscope (Olympus, Japan). The histopathological score of the colon was evaluated under a blind condition. Briefly, the histopathological score of colonic lesions was evaluated based on the degree of inflammation, lesion depth, crypt structure, etc., (Supplementary Table S3). To detect the mucous thickness and mucin-secreting goblet cells number count, AB-PAS staining was performed. AB-PAS (G1285, solarbio) staining kits were applied to the sectioned tissue slides following the manufacturers’ recommended protocols.

### Immunohistochemistry

The sections were dewaxed, rehydrated, and selected for microwave antigen retrieval. Sodium citrate buffer (PH6.0) and Tris-EDTA (PH9.0) were used for antigen retrieval. H_2_O_2_ solution was used to block endogenous peroxidase activity in colon tissues. The slides were blocked with 10% goat serum for 30 min at room temperature, and the slides were incubated with primary antibodies against occludin (OCLN) (1:100, Abcam, UK) and caspase-3 (1:200, Abcam, UK) overnight at 4°C. The second antibody was then incubated for 1 h at room temperature. Subsequently, the colon slides were visualized using DAB reagent, washed with PBS, stained with hematoxylin, and rinsed with tap water. Then the intestinal slides were dehydrated and covered. Under the microscope, four fields were randomly selected for each slide, and the color intensity was measured using Image, J.

### Western Blot Analysis

Tissues were extracted using RIPA lysis buffer with cOmplete™ protease inhibitor cocktail (4693132001, Roche, Germany). The protein concentration was measured using the Pierce™ BCA protein assay kit (23227, Thermo Fisher) according to the manufacturer’s instructions. An equivalent amount of protein (40 μg) was separated on the SDS-PAGE gel and then transferred onto the 0.45 μM PVDF membranes (Millipore, Burlington, United States) according to the standard protocols. The membranes were blocked in 5% milk with TBST buffer for 2 h at the room temperature, followed by incubation with primary antibodies (1:1000, caspase-3, 9662, CST; 1:1000, Bax, 2772, CST; 1:1000, MyD88, 4283, CST; 1:1000, NF-κB, 8242, CST; 1:1000, GAPDH, 2118, CST; 1:1000, Claudin-1, ab15089, Abcam; 1:1000,TLR5, ab62460, Abcam; 1:1000, Bcl-2, ab194583, Abcam; 1:2000, β-actin, ab8227, Abcam; 1:2000, ZO-1, 21773-1-AP, Proteintech) at 4°C overnight. After incubation with a secondary antibody for 1 h at room temperature, the proteins were detected using an ECL reagent (Millipore, Burlington, United States).

### RNA Isolation and Real-Time Quantitative RT-PCR Analysis

TRIzol reagent (15596018, Invitrogen, United States) was used to extract the total RNA from rat colon tissue. After the RNA concentration was determined using NANO DROP 2000 (Thermo Fisher, United States), reverse transcription with an Evo M-MLV Mix Kit with gDNA Clean for qPCR (AG11728, Accurate Biology), SYBR^®^ Green Premix Pro Taq HS qPCR Kit (Rox Plus) (AG11718, Accurate Biology), was used for quantitative PCR amplification. The RT-qPCR procedure was performed according to the instruction provided by the manufacturer. The results were quantified using the 2^−ΔΔCt^ method. The data obtained and the internal reference gene GAPDH were normalized and analyzed, and the sequences of primers used are listed in Supplementary Table S4.

### ELISAs

The serum levels of IL-1β (EK301B/3-96, Multisciences, China), IL-6 (EK306/3-01, Multisciences, China), TNF-α (EK382/3-02, Multisciences, China), IL-17A (EK317/3-01, Multisciences, China), IL-10 (EK310/2-01, Multisciences, China) and CRP (EK394-96, Multisciences, China) were assessed using ELISA kits. Briefly, the target protein was recognized using the capture antibody, followed by incubation with a horseradish peroxidase-conjugated secondary antibody. Thereafter, colorimetric quantification was conducted by assessing the absorbance at 450 nm using a microplate reader.

### Statistical Analysis

The biological replicates were used at least three times in all experiments. PRISM 8.01 software was used for statistical analysis and graph rendering. The results were shown as the means ± SEM. Student’s t-test and One-way ANOVA were used for statistical analysis, and *p* < 0.05 was considered statistically significant.

## Results

### SBP Ameliorates the Clinical Symptoms of TNBS-Induced Colitis

**FIGURE 1:**
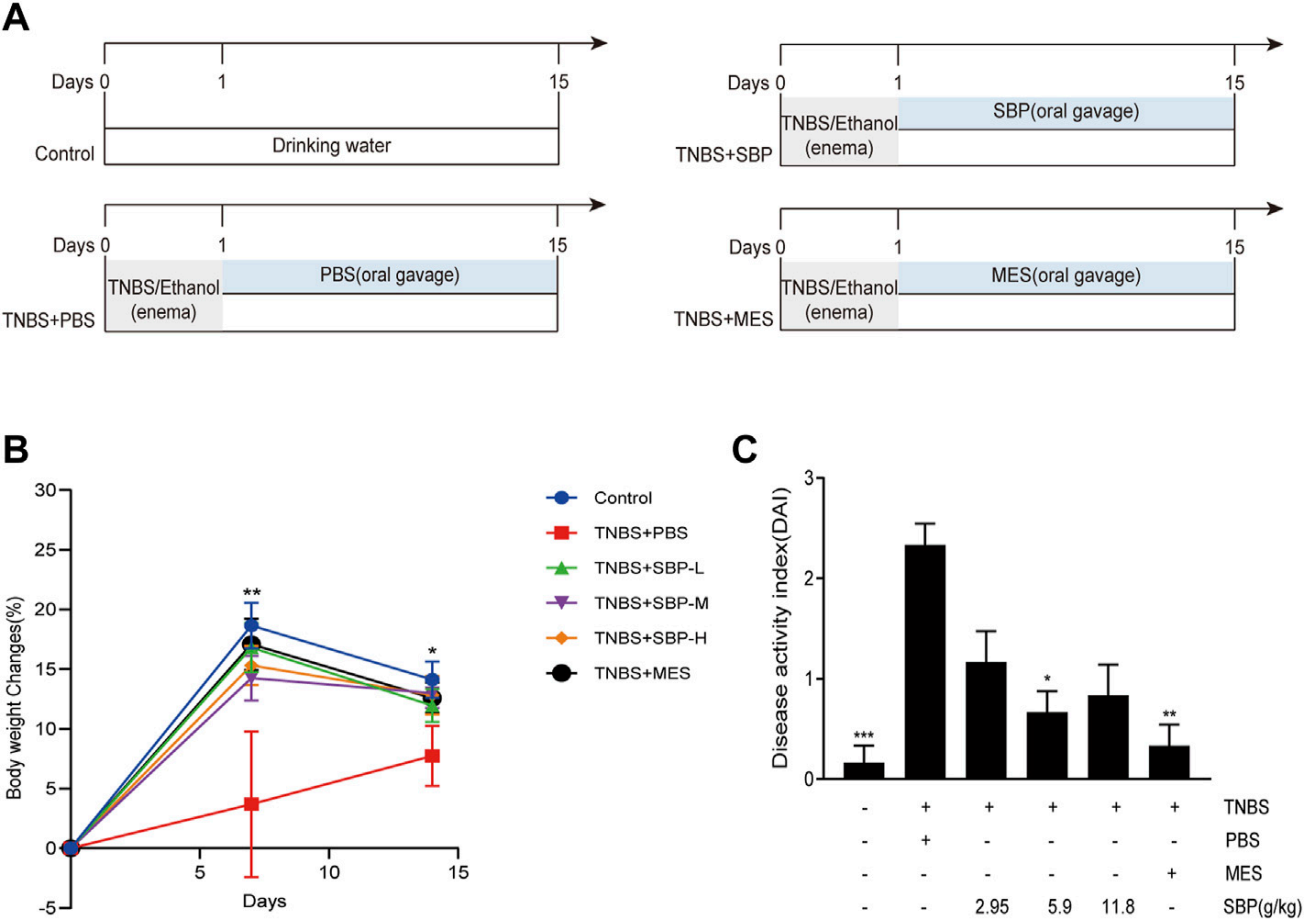
SBP intervention ameliorated the clinical signals in rats with experiment colitis. **(A)** Schematic diagram of the experimental design. **(B)** Percentage of the bodyweight change. **(C)** Disease activity index (DAI). Data shown as means ± SEM, *n* = 6 rats/group. vs. TNBS + PBS group, *p < 0.05, **p < 0.01. SBP-L, low-dose SBP group (2.95 g/kg); SBP-M, medium-dose SBP group (5.9 g/kg); SBP-H, high-dose SBP group (11.8 g/kg).

We adopted the classic TNBS/ethanol model, and the schematic of the animal experiment design is shown in Figure 1A. Compared with the control group, weight was significantly lost in TNBS-induced rats (*p* < 0.01). TNBS + SBP group (5.9 and 11.8 g/kg) had the same inhibitory effect on weight loss as TNBS + MES group (*p* < 0.05, Figure 1B). Another feature of TNBS-induced colitis was an increase in DAI. TNBS + MES and TNBS + SBP groups (5.9 g/kg) significantly reduced DAI involvement with normal stool form and no rectal bleeding compared to the TNBS + PBS group (Figure 1C).

### Effect of SBP on the Colon Length and Histopathology of Rat

**FIGURE 2:**
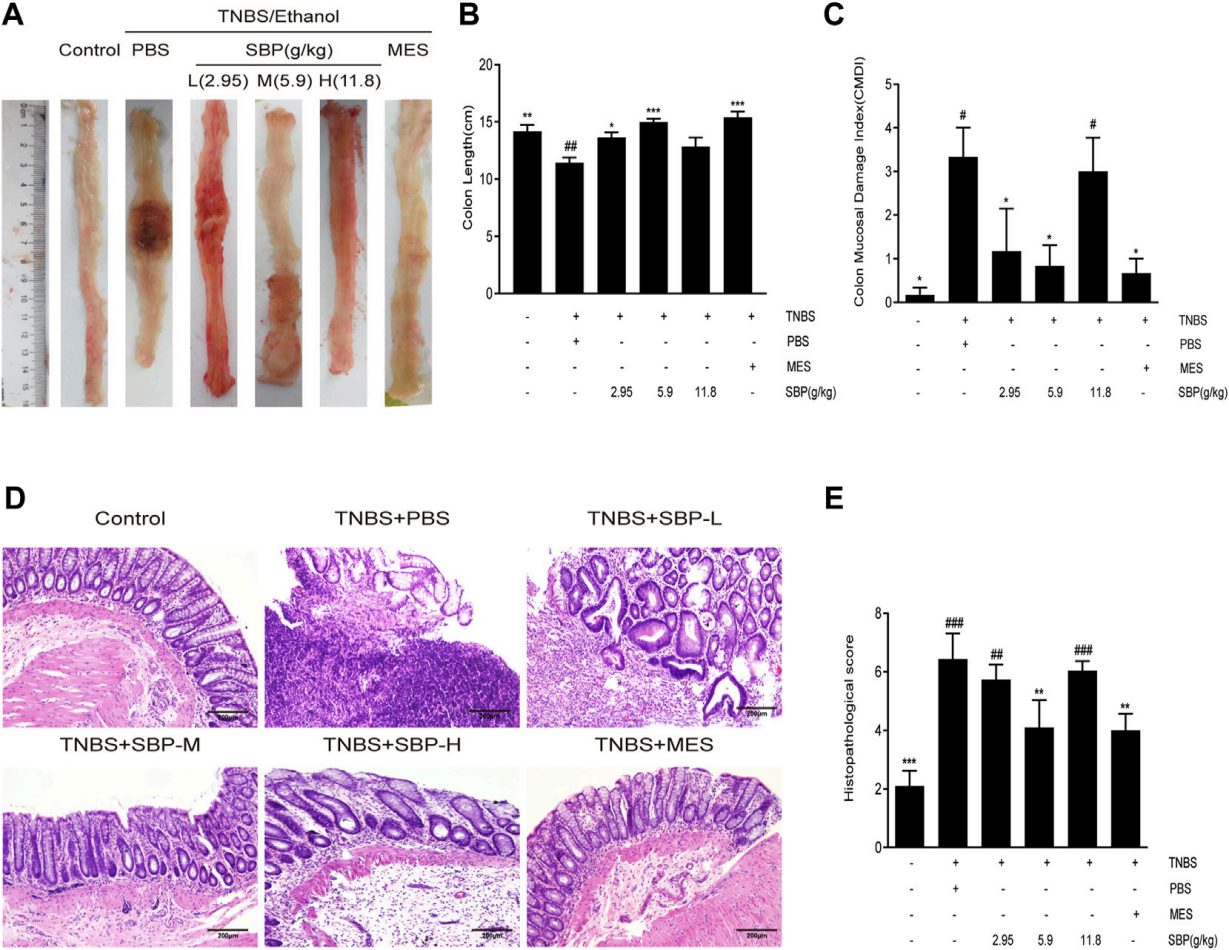
SBP on the histopathology**. (A)** Representative macroscopic colon tissue. **(B)** Colon length of each group. **(C)** Colon mucosal damage index (CMDI). **(D)** Histopathological pictures of the colon tissue using H&E staining. Scale bar = 200 µM. **(E)** Histopathological scores for colon. Data shown as means ± SEM, *n* = 6 rats/group. vs. TNBS + PBS group, *p < 0.05, **p < 0.01. vs. Control group, ^#^ *p* < 0.05, ^##^ *p* < 0.01, ^###^ *p* < 0.001.

To examine the effects of different doses of SBP on the TNBS-induced colon, we evaluated the morphology of the colon in the TNBS-induced colitis rat. The colon length was significantly shorter in TNBS-induced rats compared to the control group (*p* < 0.01). Compared with the TNBS + PBS group, the colon length was significantly increased in the SBP (2.9, 5.9 g/kg) and MES groups (Figures 2A,B). The CMDI scores showed consistent results for colon length, with significantly higher CMDI scores in the TNBS + PBS group compared with the control group. In contrast to the model group, SBP (2.8 and 5.9 g/kg) significantly reduced the CMDI scores in rats (Figure 2C). As shown in the representative image of Figure 2D and the histological score of Figure 2E, the mucosa, crypt structure, and intestinal mucosa epithelium mostly disappeared in the TNBS-induced rats, accompanied by many inflammatory cells infiltrating the submucosa and muscle layer. SBP (2.8, 11.8 g/kg) was similar to the TNBS + PBS group. The colonic damage was significantly reduced in the MES and SBP (5.9 g/kg) groups, including improved tissue architecture, reduced epithelial disintegration, and inflammatory cell infiltration. Combining the above three aspects, treatment with SBP (5.9 g/kg) significantly improved the colon length shortening and tissue damage induced by TNBS and reduced the incidence of colonic ulcers.

### SBP Suppressed Pro-Inflammatory Cytokine Expression in TNBS-Induced Colitis

**FIGURE 3:**
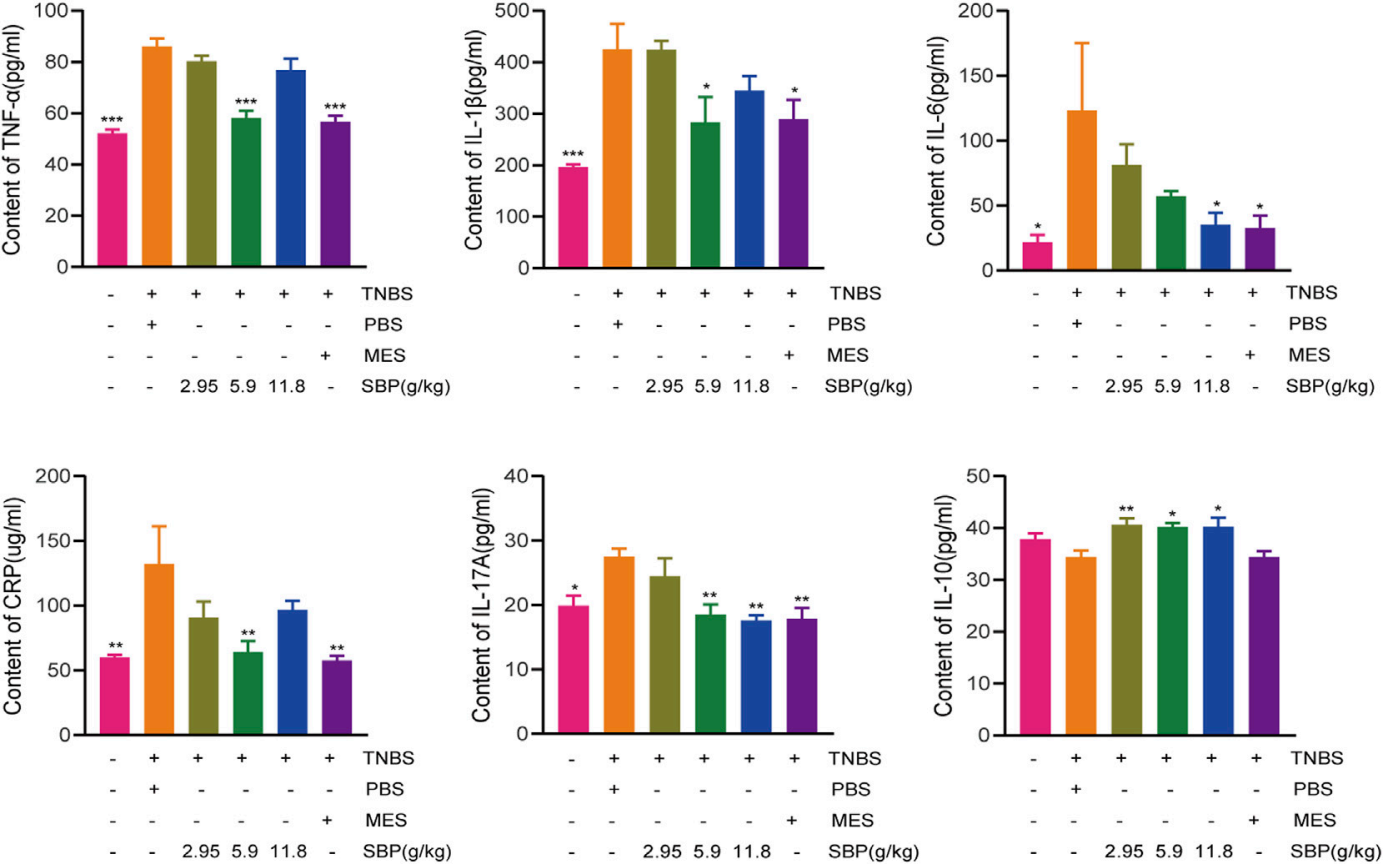
Change of cytokine after SBP treatment**. (A)**TNF-α **(B)** IL-1β **(C)** IL-6 **(D)** CRP **(E)** IL-17A and **(F)** IL-10 in serum. vs. TNBS + PBS group, *n* = 5 rats/group. Data shown as means ± SEM. *p < 0.05, **p < 0.01, ***p < 0.001.

TNBS-induced colitis usually leads to an increase in the secretion of pro-inflammatory cytokines. To evaluate the effect of SBP on pro-inflammatory response in TNBS-induced colitis rats, we analyzed the expression of inflammatory cytokines. The ELISA results showed that the expressions of CRP, IL-6, IL-17A, IL-1 β, and TNF- α in the TNBS + PBS group were significantly higher than those in the control group. Both MES and SBP (5.9 or 11.8 g/kg) treatment significantly reversed these changes. Moreover, the level of IL-10 in SBP groups (5.9 and 11.8 g/kg) were increased greatly (*p* < 0.05; *p* < 0.01). Thus, SBP shows an anti-colitis effect by inhibiting the secretion of pro-inflammatory cytokines, which are useful in maintaining the balance of inflammatory cytokines in colitis rats (Figure 3).

### SBP Inhibits Cell Apoptosis in TNBS-Induced Colitis

**FIGURE 4:**
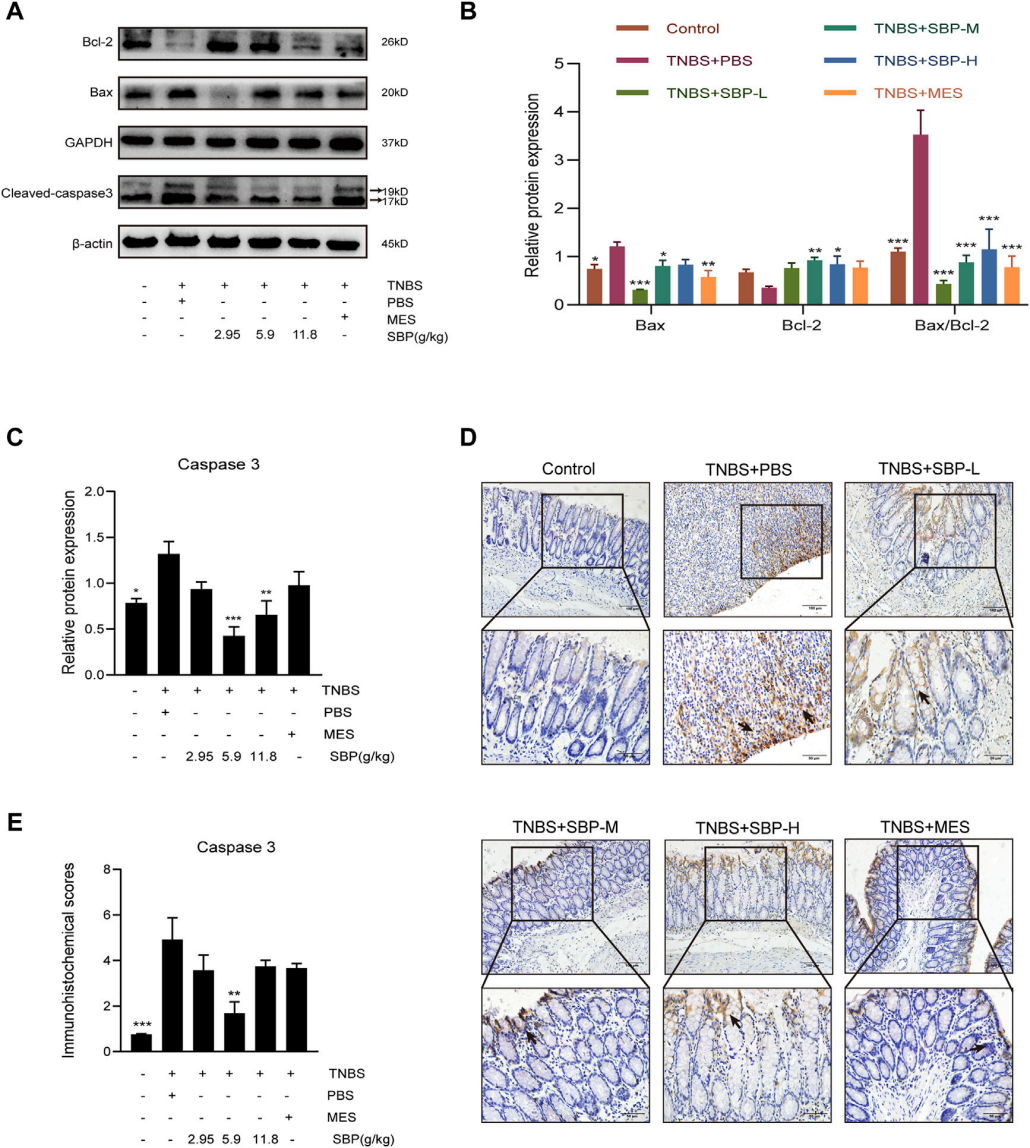
Effect of SBP on the apoptosis in TNBS-induced colitis**. (A–C)** Representative western blot analysis of Bcl-2, Bax, Cleaved-caspase 3 levels in colon tissue. **(D,E)** Immunohistochemistry analysis of cleaved caspase-3. Scale bar = 100 µM (×200); Scale bar = 50 µM (×400). vs. TNBS + PBS group, *n* = 3 rats/group. Data shown as means ± SEM. *p < 0.05, **p < 0.01, ***p < 0.001. SBP-L, low-dose SBP group (2.95 g/kg); SBP-M, medium-dose SBP group (5.9 g/kg); SBP-H, high-dose SBP group (11.8 g/kg).

The notable increase of per-apoptotic Bax and the decrease of anti-apoptotic were observed in the TNBS + PBS group. By contrast, SBP treatment augmented the expression of Bcl-2 and attenuated the expression of Bax, resulting in a significantly decreased Bax/Bcl-2 ratio (*p* < 0.001) in the colonic tissues. The ratio of Bax/Bcl-2 could reflect anti-apoptosis (Figures 4A,B). As shown in Figure 4C, the expression of cleaved-caspase-3 was upregulated in the TNBS + PBS group and downregulated in the SBP groups (especially at 5.9 g/kg). The immunohistochemistry (IHC) analysis also showed the same trend (Figure 4D,E). These results indicate that SBP positively regulates TNBS-induced colitis by blocking the damaged colon tissue.

### SBP Restored Intestinal Barrier in Experimental Colitis Models

**FIGURE 5:**
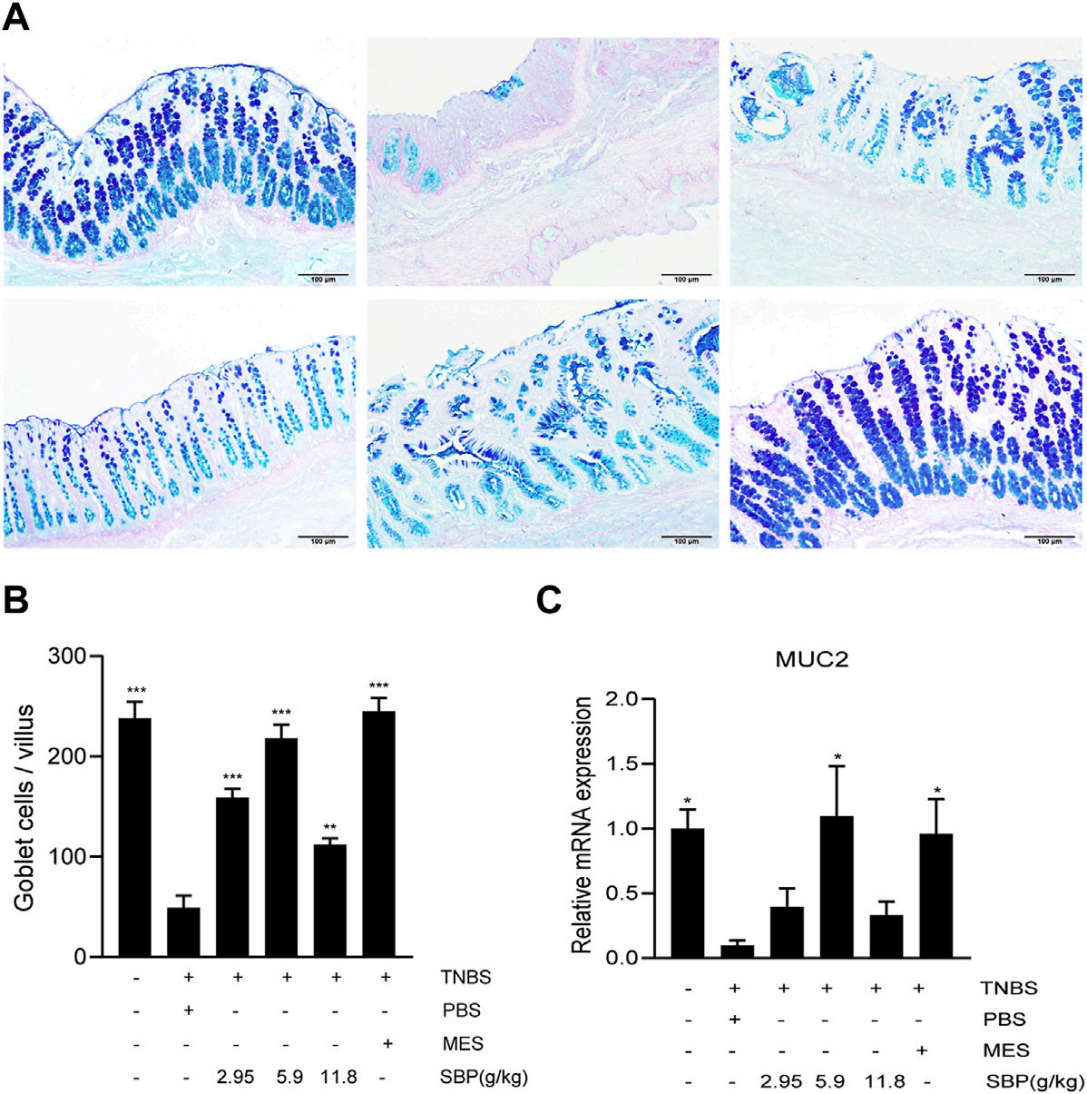
SBP restructured goblet cells and repaired the functions of disruption of the mucin barrier**. (A)** Mucin and goblet cells using AB-PAS staining. Scale bar = 100 µM. **(B)** Number of goblet cells. **(C)** mRNA expression level of MUC2 in the colon tissue was determined by qPCR. vs. TNBS + PBS group, *n* = 5 rats/group. Data shown as means ± SEM. *p < 0.05, **p < 0.01, ***p < 0.001.

The severity of colitis is positively correlated with the degree of intestinal epithelial barrier injury. Mucin is a major component of the intestinal mucous layer and provides the first intestinal barrier against pathogens. AB-PAS staining of the colon showed that the number of goblet cells in the TNBS + PBS group decreased significantly (Figure 5A), and the number of goblet cells increased after SBP (5.9 g/kg) and MES group treatment, showing a higher mucus level (Figure 5B). The expression level of MUC2 mRNA in colon tissue was significantly increased in SBP (5.9 g/kg) and MES groups than that in the TNBS + PBS group (Figure 5C).

**FIGURE 6:**
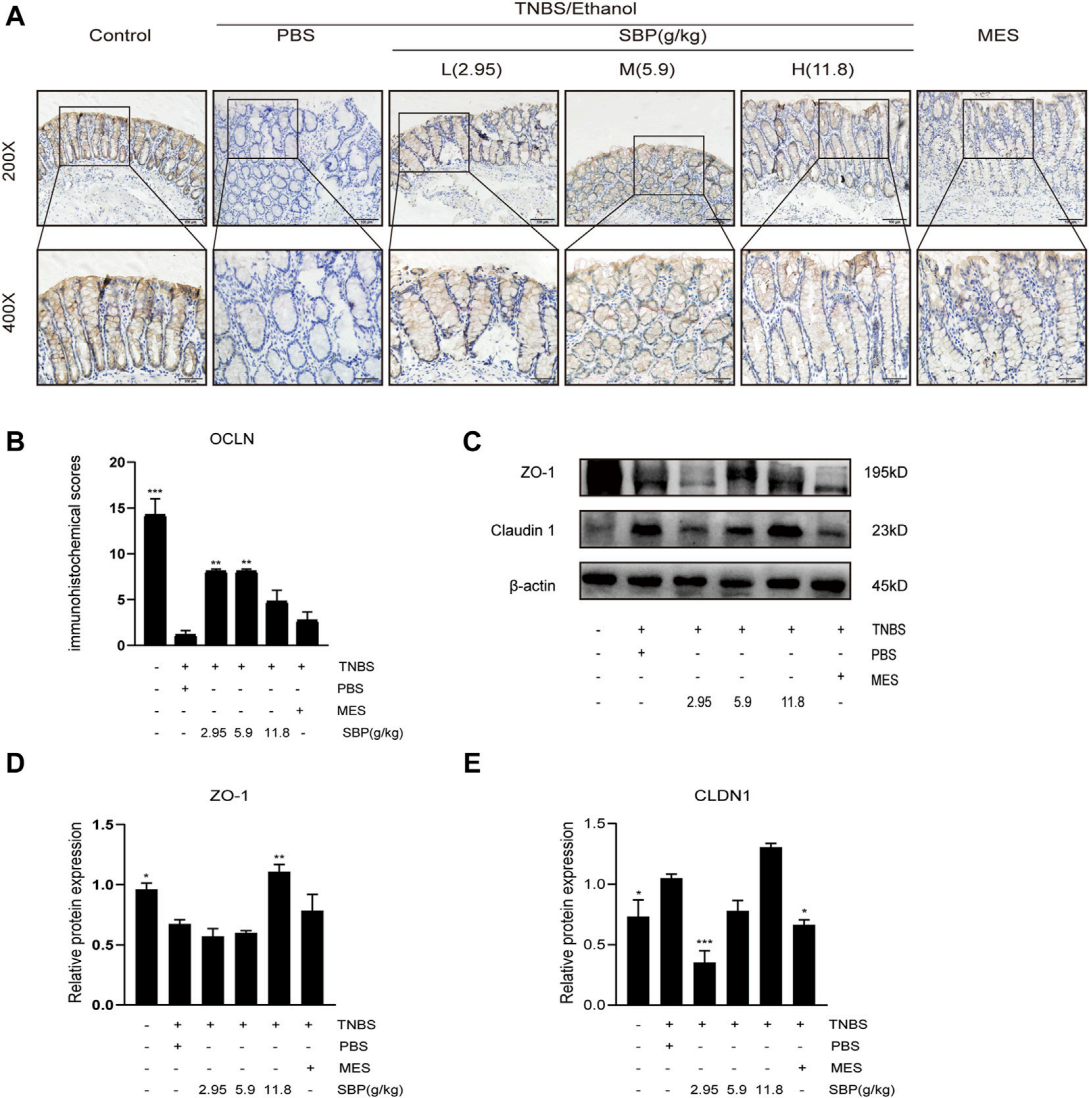
SBP restored the epithelial tight junction in TNBS-induced colitis**. (A,B)** Representative immunohistochemistry images of colon tissues stained with OCLN. Scale bar = 100 µM (×200); Scale bar = 50 µM (×400). Quantification with Fiji ImageJ. **(C–E)** Primary components of tight junction protein expression of CLDN-1 and ZO-1 in different groups. vs. TNBS + PBS group, *n* = 3 rats/group, Data shown as means ± SEM. *p < 0.05, **p < 0.01, ***p < 0.001.

TNBS-induced apoptosis of colonic epithelial cells contributes to the disruption of intestinal integrity. Therefore, we investigated the effect of SBP on the expression of the tight junction proteins using western blot (WB) and IHC analysis. Regarding the tight junction protein OCLN in IHC, the images showed that OCLN was abundant and structurally intact in the normal intestinal epithelium. The TNBS treatment disrupted OCLN distribution and localization, accompanied by crypt architectural distortions. SBP (2.95 and 5.9 g/kg) produced a protective effect, restoring OCLN expression and localization (Figures 6A,B). WB density analysis showed that SBP (11.8 g/kg) had a better effect on rebuilding the expression of ZO-1(*p* < 0.01). Compared with the TNBS + PBS group, there were no significant differences among the other treatment groups (Figures 6C,D). Compared with the control group, the protein expression of CLDN1 was significantly increased in TNBS-induced colitis and downregulated in SBP and MES treatment groups (Figures 6C,E).

### Effect of SBP on the Expression of the TLR5/MyD88/NF-κB Pathway

**FIGURE 7:**
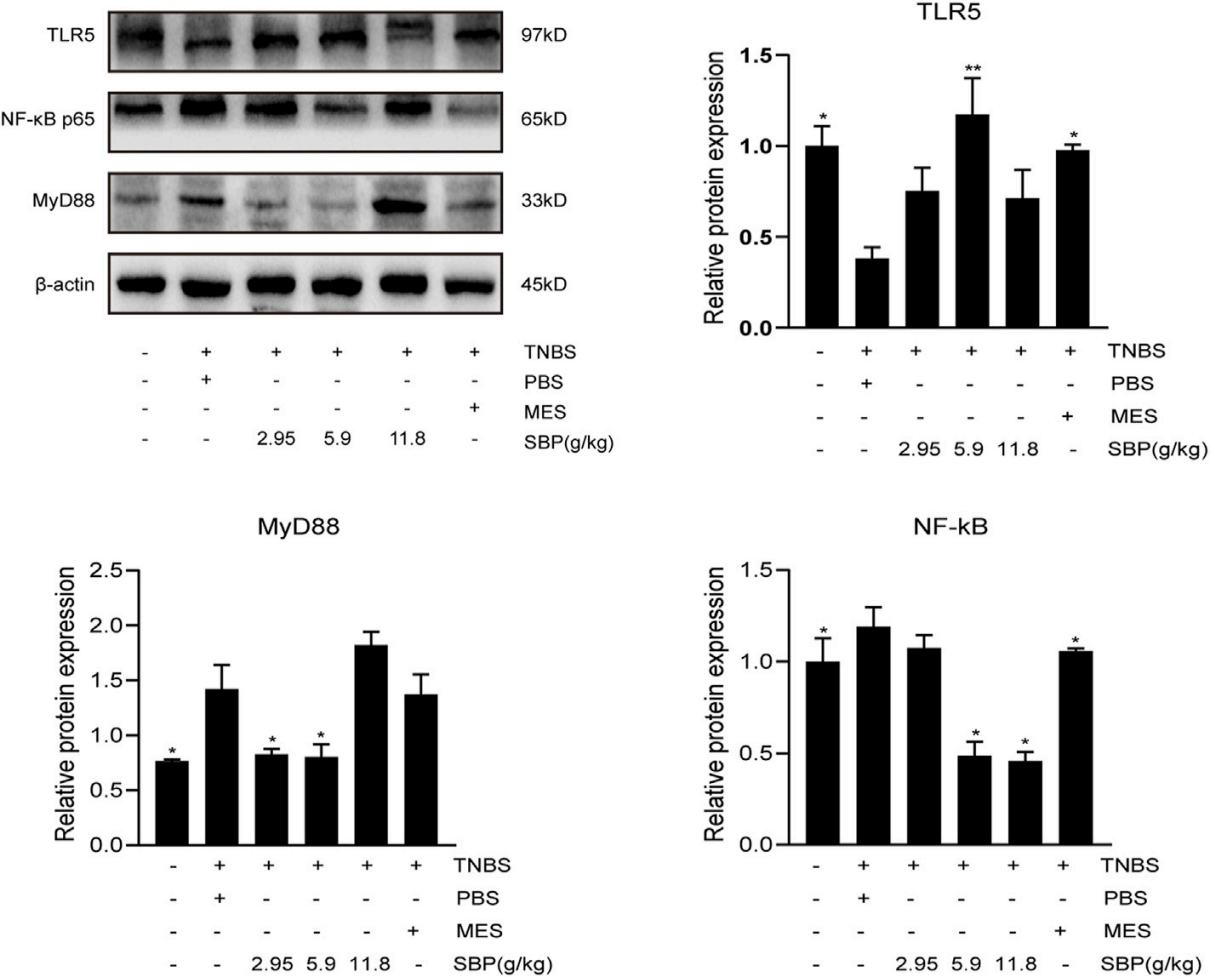
Effects of SBP on the levels of key molecules involved in the TLR5/MyD88/NF-κB signaling pathway in TNBS-induced colitis rats. The level of TLR5/MyD88/NF-κB p65 in the colonic tissues were analyzed using western blot. vs. TNBS + PBS group, *n* = 3 rats/group, Data shown as means ± SEM. *p < 0.05, **p < 0.01.

To explore the protective mechanism of SBP on TNBS-induced colitis, we detected the protein expression of TLR5 in the colon. The semi-quantitative analysis confirmed that in the TNBS-induced colitis group, there were substantial reductions in TLR5 protein levels; however, these were restored in the SBP group (5.9 g/kg) and MES group (*p* < 0.01, *p* < 0.05). The expressions of MyD88, NF-κB in the colon of rats were performed by WB. As shown in Figure 7, TNBS treatment notably increased the expression of MyD88 and NF-κB (*p* < 0.05). In comparison with TNBS + PBS group, in the SBP group (5.9 g/kg), the TNBS-induced colitis rats were treated by downregulating the expression levels of MyD88 and NF-κB (*p* < 0.05).

## Discussion

The epithelial barrier defect leads the intestinal surface to a constitutive exposure to diverse antigens, which could trigger an immunological response and promote UC, which is also known as “leaky gut” in patients with IBD (Chang et al., 2017). The leaky gut syndrome is characterized by a damaged mucin synthesis, a decreased expression of junction proteins, and epithelial cell death (Odenwald and Turner, 2013). Early in the pathogenesis of UC, increased permeability of the epithelial cells is observed to precede the clinical symptoms (Turpin et al., 2020). Thus, tightening of the intestinal epithelial barrier and suppression of inflammation are essential to induce and maintain remission of UC (Hering et al., 2012).

Many studies demonstrated that the loss of tight junction proteins is vital to the increase of intestinal epithelial barrier permeability induced by inflammatory stimuli (Zeissig et al., 2007; Bhat et al., 2019). TNBS-mediated colitis is characterized by increased inflammatory cytokine levels in the intestinal mucosa (Neurath et al., 1997). TNF-α regulates the expression of many members of tight junction proteins, such as inhibiting the activity of OCLN promoter, leading to the redistribution of ZO-1, and reducing the transepithelial electrical resistance (Mankertz et al., 2000; Wang et al., 2005; Ye and Sun, 2017), 2). Our study showed a considerable loss of OCLN and ZO-1 in the TNBS-induced colonic epithelium, accompanied by a high-level expression of caspase-3. Interestingly, the work by Kuo et al. (2019) identified that low-dose of TNF could inhibit the activity and transcription of caspase-3, leading to generalized apoptotic resistance, which might be the adaptive mechanism of enhancing epithelial survival in an inflammatory environment. However, most of the literatures reported that inflammatory cytokines partly increase the caspase-3 activity during acute injury and promote intestinal epithelial cell apoptosis by inhibiting anti-apoptotic signals such as mTORC2/AKT in the epithelial cells destroying the mucosal epithelial barrier (Arab et al., 2021; Castro-Martinez et al., 2021; Pochard et al., 2021). These studies suggest that the crosstalk between apoptosis and intestinal barrier in intestinal epithelial cells depends on the degree of inflammation. In our study, except for TNF-α, other pro-inflammatory factors such as IL-6 and IL-1β were also vastly increased in TNBS-induced colitis. The stimulation of many inflammatory factors are the leading cause of intestinal epithelial cell apoptosis. A recent study showed that ZO-1 transcript and protein expression were reduced in biopsy specimens of UC patients and caused ineffective mucosal healing in patients with IBD by regulating epithelial proliferation and mitosis (Kuo et al., 2021). In the present study, we observed the destruction of OCLN and ZO-1 in TNBS-induced colitis. Immunohistochemical analysis showed that treatment with SBP produced a protective effect and reserved the expression and localization of OCLN. These findings suggested that SBP could regulate OCLN and ZO-1 to tighten the intestinal barrier and accelerate processes of healing. Moreover, high expression of CLDN-1 protein was observed. Unlike OCLN, the study (Čužić et al., 2021) reminds that CLDN-1 alone should not be considered a tight junction protein solely involved in intestinal barrier function but also takes into account the fact that inflammatory cells express CLDN-1 in inflamed mucosa. *In vitro* and *in vivo* models, macrophages are alternatively activated by various Th2 associated and anti-inflammatory mediators expressing CLDN-1, CLDN-2, and CLDN-8 (Van den Bossche et al., 2012; Čužić et al., 2021). Weber and Pope reported that increased CLDN-1 expression in patient samples with inflammatory bowel disease is associated with early transformation stages of colitis-associated cancer. CLDN-1 regulates colonic epithelial cell differentiation in a Notch and Akt-dependent manner and promotes colitis severity by regulating MMP-9 and p-ERK signaling, while impairing colitis-associated injury/repair. CLDN-1 may be a potential biomarker for ulcerative colitis susceptibility (Weber et al., 2008; Pope et al., 2014; Gowrikumar et al., 2019). Therefore, CLDN-1 was significantly downregulated after SBP treatment, which exerted therapeutic effects by restoring tight junction structure and regulating inflammation.

In addition, even in the early stages of UC, apoptotic foci could already be observed endoscopically and result in delayed intestinal regeneration (Gitter et al., 2001; Hwang et al., 2018). We found that barrier-protective effects of SBP are associated with decreased colonic epithelial cell apoptosis. Western blot analysis was used to compare the expression of a group of apoptosis-related proteins. The dynamic balance between anti-apoptosis and pro-apoptosis was reflected by the ratio of Bcl-2/Bax (Eder et al., 2013). It was a significant decrease in the expression of cleaved-caspase3 in lamina propria of the SBP group, which correlated with a decrease in the Bax/Bcl-2 ratio. The results implied that SBP could inhibit apoptosis in the damaged colon. This effect may be related to the regulation of TLR5-mediated inflammation.

Different from other TLRs, TLR5 is highly expressed in the colonic epithelial cells (Price et al., 2018). TLR5 specifically recognizes flagellin, which is the bacterial locomotion component for detecting whether bacteria have crossed the intestinal epithelium (Vijay-Kumar and Gewirtz, 2009). It means that TLR5 is used by the intestinal mucosal immune system to detect flagellin to monitor the translocation of intestinal microbiota. Commonly, TLR5 promotes the generation of flagellin-specific IgA that serves to suppress the flagellin gene expression (Cullender et al., 2013). Similarly, humans with dominant-negative TLR5 alleles showed lower levels of naturally acquired flagellin-specific antibodies (Gewirtz et al., 2006). Decreased TLR5 expression and increased flagellin levels were found in the mucosa of IBD patients and animal models. However, they all have a common feature: the destruction of the mucous layer is colonized by many bacteria (Stanislawowski et al., 2009; Chassaing et al., 2014).

Humans have an inner mucus layer formed by MUC2 that typically separates bacteria from the epithelial cells in the intestinal mucosa. A subpopulation of colonic goblet cells recognize TLR5 ligands and elicit calcium signals that transmit through gap junctions to other goblet cells to provoke MUC2 secretion (Birchenough et al., 2016), maintaining an inner mucus layer, that is, impenetrable to bacteria and limiting the direct interaction between microbiota and epithelium. However, under continuous or repeated bacterial stimulation, MUC2 particles are constantly secreted. Subsequently, the goblet cells are gradually emptied, forming thin and small goblet cells that are not easily recognized (Johansson et al., 2014). The upregulation of partially synthesized or misfolded MUC2 induces endoplasmic reticulum stress to initiate and prolong unfolded protein response. It ultimately reduces the production of MUC2 granules, limits the refilling of MUC2 granules in goblet cells and further decreases the secretion of MUC2, aggravates mucus barrier dysfunction, and increases the exposure of colon epithelial cells to gut bacteria (Johansson et al., 2011). Due to the insufficient storage of secretory granules in goblet cells and low levels of MUC2 expression, the regulatory secretion of sentinel goblet cells is powerless in removing the invading bacteria (van der Post et al., 2019). The destruction of this mechanism might explain why the mucous layers of TLR5-/- and epithelial MUC2-deficient mice are more colonized by symbiotic microorganisms, resulting in the eventual development of spontaneous colitis. Many bacterial flagellins could reach the lamina propria of the intestinal mucosa and combine with TLR5 leading to a downregulation level of TLR5. The expression level of TLR5 was positively correlated with MUC2. Consistent with that, the present study showed that the goblet cell count was significantly reduced in the TNBS-induced colitis rats. At the same time, SBP could antagonize this effect and accelerate goblet cells reconstruction. Our results showed that the SBP could upregulate the transcription of MUC2 mRNA.

Loss of the epithelial barrier in damaged mucosa exposes TLR5 to flagellin. Once bound by activating the ligand, TLR5 could initiate a signaling cascade to trigger TLR5-mediated inflammation. It first induces the recruitment of the cytosolic adapter, MyD88, which interacts directly with the Toll-interleukin-1 receptor (TIR) domain (Akira and Takeda, 2004). The genes associated with NF-κB signaling were subsequently induced to translocate into the nucleus and induced transcription and expression of pro-inflammatory cytokines such as TNF-α, IL-6, and IL-1β (Liu et al., 2018). In the present study, the levels of IL-6, IL-17A, IL-1β, and TNF-α were increased, accompanied by increased expression of TLR5/MyD88/NF-κB pathway in TNBS induced colitis rats. The SBP could reverse these changes, which indicated that TLR5 and its related inflammatory signaling pathway might play an essential role in TNBS-induced colitis. The mechanism of SBP improving colitis may be related to the inhibition of TLR5 and its associated inflammatory pathways even it did not achieve a positive dose-effect relationship. We consider that the bias produced by the smaller sample size or the varied concentrations of the main components in the Traditional Chinese medicine formula is responsible for the different effects may be the potential reasons for the case.

**FIGURE 8:**
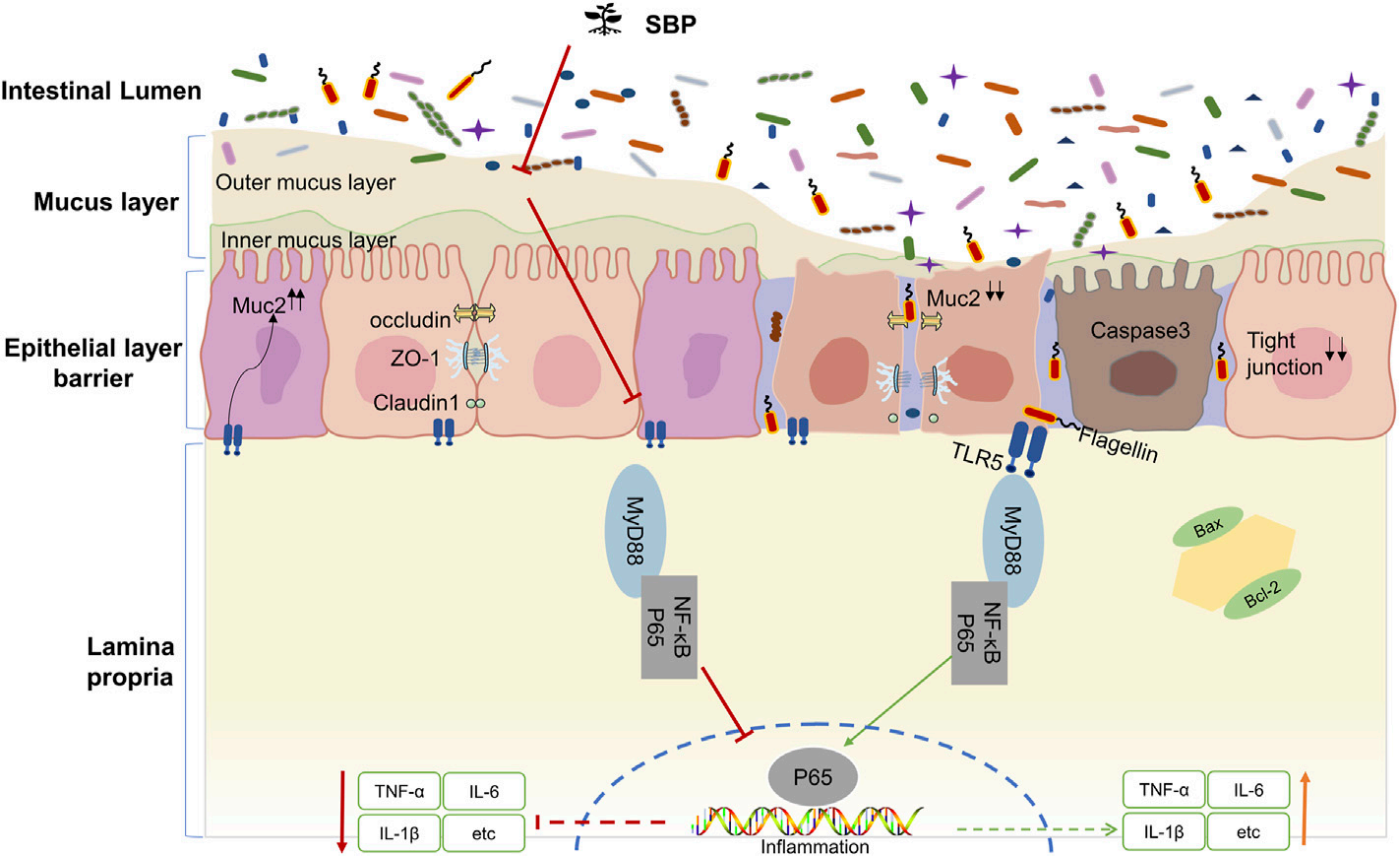
Proposed mechanism involving the TLR5/MyD88/NF-κB pathway.

In summary, our study showed that TNBS intervention could reduce TLR5 expression in colonic epithelial cells. We speculate that the mechanism by which TLR5 downregulation promotes inflammation is associated with decreased mucin secretion after goblet cell destruction, leading the bacteria to break through the mucus barrier. SBP detaches the bacteria from the intestinal crypts by reestablishing the goblet cells to repair the mucus barrier. Blocking the binding of flagellin-TLR5 resulted in two effects: 1) Increased expression levels of TLR5 in the colonic epithelium due to reduced consumption of TLR5 exert an inhibitory effect on flagellin. 2) Blocking the TLR5/MyD88/NF-κB pathway activation decreased the cytokines that signal through MyD88, such as IL-1β and IL-6, alleviated TNBS induced colitis, and maintained intestinal homeostasis. The mechanism of how SBP regulates the TLR5/MyD88/NF-κB pathway based on the present study is displayed in a schematic diagram (Figure 8).

Our study provides a basis for understanding its essential role in goblet cell physiology and the healthy developmental architecture of the mucus layer by exploring the expression and function of TLR5, a key pattern recognition receptor, in TNBS-induced colitis. The mechanism of TLR5 interaction with gut microbiota is largely unknown, and TLR5^−/−^ mice are needed to study the role of TLR5 in intestinal epithelial cells more accurately. We still lack further verification due to limited conditions, but the result is worth continuing to explore.

## Conclusion

Our results demonstrated that SBP has the potential to alleviate UC and prolong the remission stage by improving mucus protection and tightening the epithelial barrier integrity and suppressing inflammation in the host. The underlying mechanism may be related to the reconstruction of goblet cells and positive regulation of MUC2 expression, improving mucin protection, and maintaining the integrity of the intestinal barrier of the host. Meanwhile, the protective effect of SBP is achieved through limiting the activation of the TLR5/MyD88/NF-κB signaling pathway.

## Data Availability

The original contributions presented in the study are included in the article/Supplementary Material, further inquiries can be directed to the corresponding authors.
