# Supplementary material for: Shenling Baizhu Powder Alleviates TNBS-Induced Colitis in Rats by Improving Intestinal Epithelial Permeability and Inhibiting Inflammation Through the TLR5/MyD88/NF-κB Pathway
Source: Front Pharmacol. 2022 Apr 28;13:883918. doi: 10.3389/fphar.2022.883918 (PMC9096158; doi:10.3389/fphar.2022.883918)
Supplement: Supplementary file 1 [file DataSheet1.docx]

Supplementary Material

# Supplementary Tables

**Table S1 Detailed rules for DAI**

| Weight loss(%) | Stool consistency | Bleeding | Score(s) |
| --- | --- | --- | --- |
| None | Normal | Normal | 0 |
| 1-5 | —— | —— | 1 |
| 5-10 | Loose | Hemoccult positive | 2 |
| 10-15 | —— | —— | 3 |
| More than 15 | Watery diarrhoea | Gross bleeding | 4 |

DAI score =( Weight loss score + Stool consistency score + Bleeding score)/3

**Table S2 Criteria for Colon Mucosal Damage Index(CMDI)**

| Score | Criteria |
| --- | --- |
| 0 | No damage |
| 1 | Hyperaemia; no ulcers |
| 2 | Hyperaemia and thickening of the bowel wall; no ulcers |
| 3 | One ulcer without thickening of the bowel wall |
| 4 | Two or more sites of ulceration/inflammation |
| 5 | Two or more major sites of ulceration and inflammation  or one site of ulceration/inflammation extended > 1 cm  along the length of the colon |
| 6-10 | If damage covered >2 cm along the length of the colon,  the score was increased by 1 for each additional cm of  involvement |

**Table S3 The histopathological score of colonic lesions**

| Inflammation | Lesion depth | Crypt destruction | Score |
| --- | --- | --- | --- |
| None | None | None | 0 |
| Slightly | Mucosal layer | Basal 1/3 was destroyed | 1 |
| Moderate | Submucosa | Basal 2/3 was destroyed | 2 |
| Severe | Muscularis | Intact surface only | 3 |
|  | Serosa | All crypt and epithelium were destroyed | 4 |

**Table S4 Primer sets used in qPCR experiments for rats samples**

| Gene | Primer | Sequences(5’-3’) |
| --- | --- | --- |
| MUC2 | FORWARD | CTCACCAACCACCTCAACCACTTC |
|  | REVERSE | TCCAGAATCCAGCCAGCCAGTC |
| GAPDH | FORWARD | GTCCATGCCATCACTGCCACTC |
|  | REVERSE | CGCCTGCTTCACCACCTTCTTG |
